# Factors Affecting the Field Adaptation of Early-Stage Nurses in South Korea

**DOI:** 10.3390/healthcare12141447

**Published:** 2024-07-19

**Authors:** Eunhee Hwang, Miyeong Kim, Yunkyeong Lee

**Affiliations:** Department of Nursing, Wonkwang University, Iksan 54538, Republic of Korea; ehh@wku.ac.kr (E.H.); mi-young0125@naver.com (M.K.)

**Keywords:** adaptation, nurses, optimism, readiness, work environment

## Abstract

Supporting early-stage nurses to adapt to the field and become proficient in nursing is important to improve the quality of patient care. This study aimed to determine the effects of the nursing work environment, nursing practice readiness, and optimism on the field adaptation of early-stage nurses. A descriptive survey was conducted among 209 early-stage nurses with ≤3 years of work experience at hospitals. The collected data were analyzed using descriptive statistics, the *t*-test, ANOVA, Pearson’s correlation coefficients, and regression analysis with the SPSS Program. The participating nurses’ mean field adaptation score was 2.90 ± 0.40 (total score = 5) and a significant positive correlation was found between nursing work environment (r = 0.61, *p* < 0.001), nursing practice readiness (r = 0.41, *p* < 0.001), and optimism (r = 0.26, *p* < 0.001). The regression analysis revealed that the nursing work environment (β = 0.38, *p* < 0.001), job satisfaction (β = 0.33, *p* < 0.001), nursing practice readiness (β = 0.24, *p* < 0.001), and turnover intention (β = 0.17, *p* = 0.001) significantly affect the field adaptation of early stage nurses; the explanatory power was 56.1% (F = 27.55, *p* < 0.001). The results suggest that to facilitate the field adaptation of early-stage nurses, the nursing work environment, job satisfaction, and nursing practice readiness should be improved. Improvement in the nursing work environment and the development of additional training for field adaptation would enhance the ability of early-stage nurses to adapt to the field and, consequently, improve the quality of nursing care.

## 1. Introduction

### 1.1. Background

The field of nursing in South Korea has recently faced problems such as workplace bullying, suicide, and high turnover. These problems are particularly prominent among new or early-stage nurses with limited experience in hospitals [1]. It takes about 2–3 years for a new nurse to reach a competent level in performing nursing duties. Within their first three years of clinical experience [2], early-stage nurses lack practical nursing skills and field knowledge. Among hospital nurses, those with less than three years of clinical experience account for 36.6%, representing the largest proportion. However, the turnover rate within three years of employment is increasing steadily, reaching 64.1% [3]. This indicates the need to take a greater interest in the field adaptation of early-stage nurses with ≤3 years of clinical experience, as they account for 38.5% of career nurses [4].

The number of nursing graduates in South Korea is the highest among OECD countries but the number of nurses working in healthcare centers per 1000 persons is as low as 55% of the OECD average and only 47.1% of the total number of licensed nurses work in healthcare centers (*n* = 457,849 and 215,817, respectively) [5]. This implies that, in comparison to nurses in other advanced countries, nurses in South Korea do not commit themselves to long-term work in healthcare centers and leave the field soon, although the number of graduating nurses is not low [6]. The low percentage of nurses working in healthcare centers is due to the high rates of turnover and resignation and short length of service because of the high workload resulting from three-shift or night-shift work with comparatively low-quality work conditions [7]. Notably, the most common cause of turnover among nurses within a year after allocation is maladjustment in the workplace (36.5%) [4]. New graduate and early-stage nurses assume new roles and responsibilities when making the transition from student to nurse. However, the current hospital system in South Korea lacks adequate education and training to enable new nurses to perform the roles of independent nurses [8]. Hence, not only does the supply of new nurses increase the total number of nurses but an investigation of the factors affecting the field adaptation of nurses is required to provide the necessary support.

Field adaptation is a process through which a member of an organization learns the values needed for his or her assigned roles and acquires the organizational social knowledge and the ability to perform his or her roles and expected actions [9]. The experience of maladjustment in the field in terms of nursing practice could cause early turnover among new nurses [10] and subsequently lead to low levels of continuity in nursing work and healthcare service quality [11]. The same experience among early-stage nurses could lead to the same outcomes. A study on the experience of turnover intention among early stage nurses [12] found that its causes are (1) stress related to interpersonal relationships (“a conflict with a superior or coworker”, “a conflict with a patient or guardian”, and “a conflict with the healthcare team”); (2) doubts regarding the nursing profession (“lack of autonomy at work”, “lack of self-development”, “unfair treatment”, and “reduced safety measures”); (3) inability to adequately perform nursing directly (“burden of performing combined nursing tasks” and “burden of facing patient outcomes of nursing”); (4) dissatisfaction with welfare (“inadequate personal security” and “poor work conditions”); and (5) the non-nursing work-load (“burden of hospital education”, “stress of tasks outside patient care”, and “ambiguous boundaries in nursing tasks and responsibilities”). Hence, the experience of turnover intention, which promotes maladjustment in the field, appears to be determined by various factors ranging from the individual’s ability to work and manage interpersonal relationships to the individual’s perception of work and organizational characteristics. Therefore, an intervention focused on these factors will provide a way to enhance the field adaptation of nurses.

The nursing work environment is one of the organizational characteristics that support nurses in providing high-quality nursing to patients, which has been viewed as the basis of the nursing practice system [13]. The subcategories of the nursing work environment include autonomy, quality of nursing care, position and participation of nurses and managers in the organization, advancement in nursing care, opportunities for professional development, and cooperation between managers, physicians, and nurses [14]. The consequences of a poor work environment for nursing include an increase in burnout among nurses, leading toward negative outcomes such as turnover, turnover intention, and change of job [15,16,17], and high mortality and various complications in patients [18,19,20]. In contrast, a good work environment is a determinant of high-quality nursing care and it leads to high job satisfaction and a low risk of burnout among nurses [21]. Job satisfaction is considered a critical factor in preventing turnover and motivating nurses to remain in their current organization [22]. An increase in job satisfaction through an improved nursing work environment could be pursued as a way to increase the field adaptation of early-stage nurses.

Practice readiness is the concept of the attitudes and traits of a new graduate worker toward success in a given work environment [23]. The nursing practice readiness of new nurses is defined based on the attributes of cognitive and psychodynamic dimensions, such as the ability to perform nursing, understanding of the workflow, ability to make judgments of a situation, ability to prioritize tasks, and attributes of definitive dimensions, such as confidence in providing nursing care to patients, positive perception of self-worth, and stress management [24]. An inadequate level of practice readiness in new nurses could negatively affect the level of patient safety, with increased medical incidents and delayed patient recovery [25]. Hence, verifying the need for additional education and management based on an evaluation of the nursing practice readiness of early-stage nurses would lay the groundwork for enhancing the field adaptation of early-stage nurses.

Optimism is generally defined as the retaining of positive beliefs even in unfortunate situations and an optimistic individual tends to view everything in their life in a positive light and recover rapidly from the initial shock of a misfortune [26]. A study on general hospital nurses showed that a higher level of optimism could more effectively prevent the symptoms of burnout [27]. Optimism is also the most important predictor of the field adaptation of new nurses [28]. In addition to the belief that good things will happen in one’s life, an optimistic attitude to view events as arising from internal factors is crucial for the outcome of adaptation in an unfamiliar work environment.

Enhancing the nursing work performance of early-stage nurses through field adaptation in clinical practice is ultimately connected with enhanced quality of nursing care for patients. Therefore, it is necessary to investigate the varied factors that influence the clinical field adaptation of early-stage nurses and provide the respective interventions. Hence, this study aimed to identify the effects of the nursing work environment, nursing practice readiness, and optimism on the field adaptation of early-stage nurses so that the findings may serve as basic data for developing programs to enhance the field adaptation of early-stage nurses.

### 1.2. Objectives

The purpose of this study is to identify the effects of the nursing work environment, nursing practice readiness, and optimism on the field adaptation of early-stage nurses. The specific goals are as follows:

1. To analyze the general characteristics and levels of the nursing work environment, nursing practice readiness, optimism, and field adaptation in early-stage nurses;
2. To analyze the differences in the field adaptation of early-stage nurses according to the previously mentioned general characteristics;
3. To analyze the correlations between the nursing work environment, nursing practice readiness, optimism, and field adaptation in early-stage nurses;
4. To identify the factors that influence the field adaptation of early-stage nurses.

## 2. Materials and Methods

### 2.1. Study Design

This study was conducted as an online descriptive survey with convenience sampling to examine the nursing work environment, nursing practice readiness, and optimism of early-stage nurses and determine the effects of such factors on their field adaptation.

### 2.2. Participants

The study participants comprised advanced early-stage nurses with ≤3 years of clinical experience who directly care for patients, working at hospitals equivalent to or of a higher level than general hospitals including 4 hospitals in 2 regions. The optimum sample size was estimated using the G*power program. With a medium effect size of 0.15, a significance level of 0.05 for a two-sided test, and a testing power of 0.95, the estimated sample size was *n* = 204. Considering the dropout rate, the questionnaires were distributed among 221 nurses in total. After removing 12 questionnaires containing inappropriate responses, 209 questionnaires were included for final analysis.

### 2.3. Instruments

#### 2.3.1. Nursing Work Environment

The nursing work environment was assessed using the Korean version of the Practice Environment Scale of the Nursing Work Index (K-PES-NWI) [29,30], a tool developed to reflect the current situation of the nursing field in South Korea. The tool consists of 29 questions, including 4 items on adequate workforce and material support, 9 items on the basis of high-quality nursing, 9 items on the participation of nurses in hospital management, 3 items on cooperation between nurses and physicians, and 4 items on the nursing manager’s ability, leadership, and support for nurses. Each item was rated on a 5-point Likert scale ranging from 1, “Strongly disagree”, to 5, “Strongly agree”, and a higher score indicated a more positive perception of the nursing work environment. The Cronbach’s α value was 0.93 in Cho et al. [30] and 0.92 in this study.

#### 2.3.2. Nursing Practice Readiness

Nursing practice readiness was measured using a tool developed by Kim [31]. The tool comprises 35 questions, including 16 items on clinical judgment and nursing performance, 8 items on professional values and attitudes, 5 items on patient-centered attitudes, 3 items on self-control, and 3 items on cooperative interpersonal relationships. Hence, this tool constitutes five categories. Each item was rated on a 4-point Likert scale. The Cronbach’s α value was 0.90 in Kim [31] and 0.87 in this study.

#### 2.3.3. Optimism

Optimism was measured using the Korean version of the Life Orientation Test-Revised (K-LOT-R). The original LOT tool was developed by Scheier and Carver [32] and the revised LOT-R tool was developed by Scheier et al. [33]. The translated tool, K-LOT-R, was developed by Shin [34]. The tool consists of 10 questions, including 3 positive and 3 negative items regarding general expectations for an optimistic life and 4 control questions to prevent participants from guessing the purpose of the questionnaire. Each item was rated on a 5-point Likert scale. The scores of the misleading questions were excluded and the negative items were reverse-scored. A higher total score indicates a higher level of optimism. The Cronbach’s α value was 0.72 in Shin [34] and 0.68 in this study.

#### 2.3.4. Field Adaptation

Field adaptation was measured using a tool developed by Son et al. [9]. The tool comprises 39 questions across 7 categories (personal traits, group characteristics, career identity, job performance, job satisfaction, commitment, and burnout). Each item was rated on a 5-point Likert scale ranging from 1, “Strongly agree”, to 5, “Strongly disagree”, and higher scores indicated higher levels of field adaptation. The Cronbach’s α value was 0.98 in Son et al. [9] and 0.87 in this study.

### 2.4. Data Analysis

The collected data were analyzed using the SPSS/WIN 26.0 program. Descriptive statistics were obtained for general characteristics, nursing work environment, nursing practice readiness, optimism, and field adaptation. A *t*-test, ANOVA, and Scheffé post hoc test were performed to analyze the differences in field adaptation according to the general characteristics. To analyze the correlations between the nursing work environment, nursing practice readiness, optimism, and field adaptation, Pearson’s correlation coefficients were used. To determine the effects of the nursing work environment, nursing practice readiness, and optimism on field adaptation, hierarchical regression analysis was used.

### 2.5. Ethitcal Considerations and Data Collection

This study was conducted with the approval of the Institutional Review Board (IRB) of the author’s institution (IRB No. WKIRB-202204-SB-031). Prior to data collection, the study purpose, methods, anonymity measures, and privacy protection considerations were explained to the manager of each investigated center. For data collection, an online Google questionnaire form was used. Google Forms is a commonly used tool for various online surveys, designed to be easily accessible to both researchers and respondents. Based on recommendations from the department manager regarding early-stage nurses who meet the included criteria, Google questionnaire links were individually sent to them through social media to request a response. The first page of the questionnaire presented the study purpose, agreement on personal data collection, and principles of privacy protection to enable eligible nurses to voluntarily agree to participate. The participants’ responses to the survey were automatically coded to remove personal identification via the computerized system. The data collection period was from 16 May to 28 October 2022.

## 3. Results

### 3.1. General Characteristics of Participants

The mean age of the participants was 24.90 ± 2.22 years, with 198 nurses in their 20s (94.7%). Regarding sex, there were 187 female nurses (89.5%). Most of the participants lived with family (*n* = 102, 48.8%). Participants living alone (*n* = 97, 46.4%) comprised the second highest number, followed by those living with a friend and senior or junior coworker (*n* = 10, 4.8%). Regarding current institutions, 165 participants worked at tertiary general hospitals (78.9%) and 44 in general hospitals (21.1%). The average total clinical experience was 14.14 ± 9.79 months and the majority of nurses had ≤12 months of experience (*n* = 106, 50.7%). In terms of the department, 116 nurses (55.5%) worked in special wards and others, 49 nurses (23.4%) worked in general wards, and 44 nurses (21.1%) worked in surgical wards. Furthermore, 194 (92.8%) worked in shifts and 127 nurses (60.8%) were allocated to their departments of choice. Most of the participants (*n* = 120, 57.4%) reported a “moderate” level of satisfaction with their current job. In all, 108 nurses (51.7%) reported the subjective health status of their health as “moderate” and 63 nurses (30.1%) reported the subjective health status of their health as “poor”. A total of 151 participants (72.2%) expressed turnover intention (Table 1).

### 3.2. Nursing Work Environment, Nursing Practice Readiness, Optimism, and Field Adaptation

The mean score for the nursing work environment was 2.57 ± 0.42 (total score = 4). Among the subcategories of the nursing work environment, the basis of high-quality nursing scored highest at 2.84 ± 0.42, whereas adequate workforce and material support scored lowest at 2.11 ± 0.62. The mean score for nursing practice readiness was 3.01 ± 0.30 (total score = 4). Among the subcategories of nursing practice readiness, self-control scored highest at 3.27 ± 0.42, while professional values and attitudes scored lowest at 2.75 ± 0.45. The mean score for optimism was 3.29 ± 0.57 (total score = 5). The mean score for field adaptation was 2.90 ± 0.40 (total score = 5); personal traits scored the highest among the subcategories (3.70 ± 0.50), while commitment scored the lowest (2.24 ± 0.69) (Table 2).

### 3.3. Differences in Field Adaptation According to General Characteristics

The level of field adaptation among the participants varied significantly according to the type of current institution (t = 2.11, *p* = 0.036), shift work (t = −2.20, *p* = 0.029), satisfaction with the current job (F = 48.94, *p* < 0.001), subjective health status (F = 17.93, *p* < 0.001), and turnover intention (t = −8.83, *p* < 0.001). The level of field adaptation was higher among nurses working at tertiary general hospitals than those at general hospitals. It was also higher among nurses who did not work in shifts and those who did not express turnover intention. The post-hoc test indicated that the level of field adaptation was significantly higher among nurses who were adequately satisfied with their current job than those who were moderately satisfied or dissatisfied with their current job. Nurses with good subjective health status also showed higher levels of field adaptation than those with moderate or poor subjective health status (Table 3).

### 3.4. Correlations between Nursing Work Environment, Optimism, Nursing Practice Readiness, and Field Adaptation

Correlation analysis on the participants’ nursing work environment, optimism, nursing practice readiness, and field adaptation indicated that field adaptation is positively and significantly correlated with the nursing work environment (r = 0.61, *p* < 0.001), nursing practice readiness (r = 0.41, *p* < 0.001), and optimism (r = 0.26, *p* < 0.001) (Table 4). A positive correlation was also found between optimism and nursing practice readiness (r = 0.35, *p* < 0.001) and between nursing practice readiness and nursing work environment (r = 0.16, *p* = 0.020).

### 3.5. The Influencing Factors of Field Adaptation

To identify the factors that affect the field adaptation of participating nurses, hierarchical multiple regression analysis was performed (Table 5). The factors that were associated with significant variation in field adaptation, namely, current institution type, shift work, satisfaction with the current job, subjective health status, and turnover intention, were treated as dummy variables for the analysis. The Durbin–Watson value was 1.74 (close to 2), indicating no problem in terms of autocorrelation or independence of the error terms. The Variance Inflation Factors (VIF) ranged between 1.04 and 2.79, thus meeting the criteria of ≤10, and the tolerance ranged between 0.36 and 0.93, indicating the lack of multicollinearity among independent variables.

Model I tested the effects of the participants’ general characteristics on field adaptation and the result indicated that the field adaptation of nurses adequately satisfied (β = 0.52, *p* < 0.001) or moderately satisfied (β = 0.25, *p* = 0.002) with their job was higher than that of those dissatisfied with their current job. It was also higher among nurses with good subjective health status (β = 0.14, *p* = 0.042) and nurses without turnover intention (β = 0.28, *p* < 0.001). The overall explanatory power of these variables for field adaptation was 39.9% (F = 20.77, *p* < 0.001).

Model II added the independent variables, namely, nursing work environment, nursing practice readiness, and optimism, to Model I; the result indicated that the field adaptation of nurses who were adequately satisfied (β = 0.33, *p* < 0.001) or moderately satisfied (β = 0.15, *p* = 0.027) with their job was higher than that of nurses dissatisfied with their current job compared to nurses without turnover intention (β = 0.17, *p* = 0.001). Additionally, the nursing work environment (β = 0.38, *p* < 0.001) and nursing practice readiness (β = 0.24, *p* < 0.001) had significant effects on the field adaptation of early-stage nurses and the overall explanatory power of these variables for field adaptation was 56.1% (F = 27.55, *p* < 0.001).

## 4. Discussion

Enabling early-stage nurses to adapt to the field and grow professionally is critical as a process to enhance the quality of nursing care. This study was conducted to identify the factors that affect the field adaptation of early-stage nurses to provide basic data for developing various intervention programs.

The level of field adaptation of the nurses who participated in this study was 2.90 out of 5. This result concurs with the findings of a previous study that applied the same instrument to measure the field adaptation of new nurses working at hospitals equivalent to or at a higher level than general hospitals (2.98) [28] but exceeds the level measured for new nurses in a different study (2.79) [35]. This variation is presumably because the participants in the previous studies were new nurses with <1 year of clinical experience, while those in this study were early-stage nurses with ≤3 years of clinical experience. In other words, compared with new nurses with <1 year of clinical experience, early-stage nurses with ≤3 years of clinical experience are likely to show relatively higher levels of field adaptation as a result of their longer duration of performing work in the clinical field. In addition, new nurses characteristically display fear stemming from their lack of experience in new situations, such as a national disaster [36]. Considering that this study was conducted during the COVID-19 pandemic, the results were presumably influenced by the nurses having to perform work in compliance with rapidly changing national policies as well as adapting to the field of nursing. Therefore, continuous follow-up studies should investigate the field adaptation of early-stage nurses. The results of this study revealed that, among the subcategories of field adaptation, “personal traits” scored the highest whereas “commitment” scored the lowest. Commitment is a positive response toward the organization and a higher level of commitment leads to a strong sense of membership as the individual strives to achieve advancement with an increased drive for work [37]. Commitment plays an important role in increasing productivity and validity [38,39] and according to Jeon [40], who reported the association of a higher level of commitment with a higher level of organizational socialization, i.e., field adaptation, hospital policies should be developed to increase the commitment of early stage nurses to enhance their field adaptation.

A nursing work environment score of ≥2.5 can be taken to indicate favorable nursing work conditions, whereas a score of <2.5 can be taken to indicate poor conditions [29]. In this study, the mean nursing work environment score was 2.57 out of 4, suggesting that most nurses perceived their nursing work environment positively. This score is lower than the 3.06 obtained in the study by Lee and Suh [41], wherein the nursing work environment was measured using the same instrument. However, it is higher than the 2.39 of the study by Kwon and Kim [15] on new nurses in small to mid-size hospitals. This implies a variation in the nursing work environment according to hospital size. Among the subcategories of nursing work environment, “adequate workforce and material support” scored the lowest, aligning with a previous study [42]. The nursing work environment has an effect on the turnover of early-stage nurses [43]. Moreover, these nurses show low motivation regarding long-term service at a hospital with high turnover intention, as they characteristically decide on turnover soon after joining the hospital [44]. Additionally, the inevitable shortage in public healthcare personnel, which includes the shortage caused by nurse resignations due to the outbreak of infectious diseases such as the COVID-19 pandemic [45], suggests the necessity of support and institutional strategies for the improvement in the nursing work environment through measures such as increased nursing workforce and work assignment in accordance with nurse preferences.

The nursing practice readiness score of early-stage nurses was 3.01 out of 4, at a moderate or higher level. Among the subcategories of nursing practice readiness, “professional values and attitudes” and “clinical judgment and nursing performance” scored low. Despite being exposed to various psychological burdens associated with the performance of nursing work [31], early-stage nurses should exhibit the right values and attitudes as health professionals for suitable clinical judgment and nursing care. However, it is characteristic of early-stage nurses to have an underdeveloped capacity to solve clinical issues independently and their competence is limited in situations that necessitate help from a coworker [10]. Coworker support is therefore crucial in reducing the burdens and challenges early-stage nurses encounter in providing nursing care in an unfamiliar environment. Through coworker support, early-stage nurses can increase the stability of their nursing performance [46,47]. Hence, a suitable support system and educational programs on clinical practice should be developed to enhance nursing practice readiness among early-stage nurses.

The participants in this study scored 3.29 out of 5 for optimism, lower than the 3.61 obtained in a previous study on clinical nurses, wherein optimism was measured using the same instrument [48]. The observably lower level of optimism among early-stage nurses when compared with experienced nurses [49] accounts for the deviation in this study on early-stage nurses. Optimism plays a positive role in improving nursing work performance [50]. With optimism, nurses can anticipate a positive future and evaluate the significance of the present in a positive light, thereby building the self-confidence to efficiently deal with the myriad situations that may arise in daily work [51]. As optimism can be cultured through positive exploratory activities [52], various ways should be developed to increase optimism among early-stage nurses.

The field adaptation of the participants in this study was analyzed according to the general characteristics and the result showed that nurses at tertiary general hospitals had higher levels of field adaptation than those at general hospitals. Nurse recruitment was shown to be relatively more difficult in general hospitals than in tertiary general hospitals [15] and “adequate workforce” scored the lowest among the subcategories of nursing work environments in this study. This suggests that the difference in field adaptation between nurses at tertiary general hospitals and those at general hospitals is likely due to the difference in nursing workforce support. According to Kang and Kang [35], field adaptation improved with increased job satisfaction, which is in line with the findings of this study. The factors that affect job satisfaction include coworker support, personal factors, environmental factors, and work-life balance; as these factors are considered crucial to increasing the job satisfaction of early-stage nurses [47,53], they should be adequately reflected in the analysis. The results of this study revealed that nurses who did not work in shifts and had good subjective health status scored high in field adaptation. Shift work may disrupt the natural biorhythm of nurses and cause physical problems, which could negatively affect health and nursing work [54]. The health of an early-stage nurse could be jeopardized if the nurse must work in shifts in addition to performing unfamiliar tasks. While shift work is unavoidable for nurses, organizational support should be provided to ensure that early-stage nurses maintain good health as they learn nursing work.

Analysis of the correlation between field adaptation and nursing work environment, nursing practice readiness, and optimism showed that the tested variables were positively and significantly correlated with the field adaptation of early-stage nurses. The results of this study demonstrated that field adaptation increased as the positive perception regarding the nursing work environment increased. This finding is in line with a previous study that reported that the nursing work environment had an effect on the active service and field adaptation of nurses [55]. This result also aligns with the findings of Kim (2020b) [31], which emphasized the role of nursing practice readiness in facilitating the successful adaptation of early-stage nurses to the field of nursing. The results of this study also revealed that field adaptation increased as optimism increased, in concurrence with Seo [56], who reported that field adaptation increased as positive psychological capital increased.

Factors that affect the field adaptation of early-stage nurses were identified in this study and the results indicate that the significant influencing factors are nursing work environment, job satisfaction, nursing practice readiness, and turnover intention, with the effect of nursing work environment being the strongest. Various factors play a role in terms of the nursing work environment: system operation and efficiency within the organization play crucial roles [57], while stress and turnover intention in nurses increase in poor work conditions [43]. The subcategories of the nursing work environment include autonomy, quality of nursing care, position and participation of nurses and managers in the organization, advancement in nursing care, opportunities for professional development, and cooperation between managers, physicians, and nurses [14]. To create a positive nursing work environment, it is necessary to promote a culture in which nurses can work with autonomy, provide support for high-quality nursing performance, invest in the personal development of nurses, and establish an organizational culture oriented toward continuous cooperation between physicians and nurses. Such efforts and changes would contribute toward reducing the turnover rate of nurses and enhancing the quality of nursing care for patients.

The following recommendations are made based on the findings of this study. First, the nursing work environment was identified as an important variable in this study. It is necessary to further explore the various factors that constitute the nursing work environment and conduct an in-depth analysis of the association of these factors with the field adaptation of nurses. Second, this study was conducted among early-stage nurses with ≤3 years of clinical experience. Therefore, future studies should investigate a wider scope of participants, including returning nurses, nurses who have changed departments, and nurses returning after parental leave, all of whom require efficient field adaptation. Third, a qualitative study to understand the experience of the field adaptation of early-stage nurses should be conducted. Fourth, studies to develop and validate intervention programs based on the influencing factors of field adaptation should be conducted, whereby the characteristics of each healthcare center should be reflected.

This study has several limitations. First, this study was conducted among early-stage nurses working at hospitals equivalent to or at a higher level than general hospitals; it was conducted during the COVID-19 pandemic, which prevents the generalization of the results to all early-stage nurses. Second, since this study focused on early-stage nurses employed in specific hospitals, careful consideration is warranted when interpreting the generalizability of the research findings. Third, although both organizational resources and authentic leadership play a critical role in early career burnout development over time [58], this study does not consider these factors, which limits the interpretation of factors affecting the field adaption of early-stage nurses. Nevertheless, this study is significant in having identified the level of field adaptation in early-stage nurses with ≤3 years of clinical experience as well as the associated influencing factors, based on which significant insights could be offered toward improving the field adaptation of early-stage nurses.

## 5. Conclusions

With natural disasters such as the COVID-19 pandemic, the problem of reduced nursing workforce has increased and the role of early-stage nurses has been discussed with emphasis when seeking solutions. However, early-stage nurses understandably take a substantial amount of time to adapt to the clinical field and the stability of field adaptation relies on the nurses performing diverse roles. This study was conducted for the field adaptation of early stage nurses with such characteristics to identify the influencing factors to ensure that the nurses mature into expert health professionals and that the quality of nursing care is enhanced.

The results of this study indicate that the field adaptation of early-stage nurses is at a moderate or higher level, with significant positive correlations between the nursing work environment, nursing practice readiness, and optimism. The field adaptation of early-stage nurses was also shown to be influenced by the nursing work environment, job satisfaction, nursing practice readiness, and turnover intention. It is necessary to foster a workplace environment that enhances job satisfaction on an organizational level and provide tailored education to improve nursing practice readiness, thereby ultimately lowering the intention to turnover for early-stage nurses. Thus, the field adaptation of early-stage nurses can be enhanced and consequently, high-quality nursing service can be expected.

## Figures and Tables

**Table 1:** Participants’ general characteristics (*N* = 209).

Characteristic	Category	n (%)	M (SD)
Age	20’s	198 (94.7)	24.90 (2.22)
(Years)	30’s	11 (5.3)	
Gender	Male	22 (10.5)	
	Female	187 (89.5)	
Living	With family	102 (48.8)	
	Alone	97 (46.4)	
	With friends, seniors and juniors	10 (4.8)	
Current institution type	Tertiary general hospital	165 (78.9)	
	General hospital	44 (21.1)	
Clinical experience	≤12	106 (50.7)	14.14 (9.79)
(month)	13 ≤ 24	68 (32.5)	
	25 ≤ 36	35 (16.7)	
Department	Special ward and others	116 (55.5)	
	general ward	49 (23.4)	
	Surgical ward	44 (21.1)	
Shift work	Yes	194 (92.8)	
	No	15 (7.2)	
Allocated department	Yes	127 (60.8)	
	No	82 (39.2)	
Satisfaction with current job	Adequately Satisfaction	53 (25.4)	
	Moderate	120 (57.4)	
	Dissatisfaction	36 (17.2)	
Subjective health status	Good	38 (18.2)	
	Moderate	108 (51.7)	
	Poor	63 (30.1)	
Turnover intention	Yes	151 (72.2)	
	No	58 (27.8)	

M = Mean, SD = Standard deviation.

**Table 2:** Level of the nursing work environment, nursing practice readiness, optimism, and field adaptation (*N* = 209).

Variable	Min	Max	M (SD)	Items	Range
Nursing work environment	1.17	3.76	2.57 (0.42)	29	1~4
Adequate workforce and material support	1.00	3.75	2.11 (0.62)	4	1~4
Basis of high-quality nursing	1.33	3.89	2.84 (0.42)	9	1~4
Participation of nurses in hospital operation	1.00	3.67	2.42 (0.49)	9	1~4
Cooperation between nurses and doctors	1.00	4.00	2.67 (0.58)	3	1~4
Nursing manager skills, leadership, and support for nurses	1.00	4.00	2.69 (0.57)	4	1~4
Nursing practice readiness	1.74	3.97	3.01 (0.30)	35	1~4
Clinical judgment and nursing performance	1.63	4.00	2.99 (0.33)	16	1~4
Professional values and attitudes	1.00	4.00	2.75 (0.45)	8	1~4
Patient-centered attitude	2.00	4.00	3.25 (0.42)	5	1~4
Self-control	1.67	4.00	3.27 (0.42)	3	1~4
Cooperative interpersonal relationships	1.67	4.00	3.16 (0.41)	3	1~4
Optimism	1.50	4.83	3.29 (0.57)	6	1~5
Field adaptation	1.59	3.95	2.90 (0.40)	39	1~5
Personal traits	2.13	5.00	3.70 (0.50)	8	1~5
Organization	1.38	4.50	2.93 (0.56)	8	1~5
Occupational identity	1.00	5.00	3.36 (0.73)	3	1~5
Performance of duties	1.00	4.80	2.58 (0.78)	5	1~5
Job satisfaction	1.40	4.40	2.91 (0.57)	5	1~5
Commitment	1.00	4.40	2.24 (0.69)	5	1~5
Exhaust	1.00	4.60	2.26 (0.73)	5	1~5

M = Mean, SD = Standard deviation.

**Table 3:** Field adaptation according to general characteristics (*N* = 209).

Characteristic	Category	M (SD)	t/F (*p*)	Scheffé
Age	20’s	2.89 (0.40)	−1.18 (0.261)	
(Years)	30’s	2.99 (0.26)		
Gender	Male	2.94 (0.41)	0.48 (0.629)	
	Female	2.89 (0.40)		
Living	With family	2.91 (0.42)	0.19 (0.828)	
	Alone	2.89 (0.36)		
	With friends, seniors, and juniors	2.83 (0.53)		
Current institution type	Tetiary general hospital	2.93 (0.38)	2.11 (0.036)	
	General hospital	2.79 (0.44)		
Clinical experience	≤12	2.91 (0.42)	1.47 (0.232)	
(month)	13 ≤ 24	2.84 (0.39)		
	25 ≤ 36	2.98 (0.33)		
Department	Special ward and others	2.95 (0.41)	2.02 (0.135)	
	General ward	2.84 (0.37)		
	Surgical ward	2.83 (0.38)		
Shift work	Yes	2.88 (0.40)	−2.20 (0.029)	
	No	3.11 (0.35)		
Allocated department	Yes	2.94 (0.41)	1.75 (0.081)	
	No	2.84 (0.37)		
Satisfaction with current job	Adequate satisfaction ^a^	3.23 (0.28)	48.94 (<0.001)	c < b < a
	Moderate ^b^	2.85 (0.35)		
	Dissatisfaction ^c^	2.55 (0.32)		
subjective health status	Good ^a^	3.17 (0.37)	17.93 (<0.001)	c < b < a
	Moderate ^b^	2.91 (0.36)		
	Poor ^c^	2.71 (0.39)		
Turnover intention	Yes	2.78 (0.38)	−8.83 (<0.001)	
	No	3.20 (0.27)		

^a,b,c^ subgroups classified based on the mean values of field adaptation.

**Table 4:** Correlation between variables (*N* = 209).

	Nurising Work Environment	Nursing Practice Readiness	Optimism	Field Adaptation
	r (*p*)	r (*p*)	r (*p*)	r (*p*)
Nursing work environment	1			
Nursing practice readiness	0.16 (0.020)	1		
Optimism	0.09 (0.216)	0.35 (<0.001)	1	
Field adaptation	0.61(<0.001)	0.41 (<0.001)	0.26 (<0.001)	1

**Table 5:** Correlation between variables (*N* = 209).

Variables	Field Adaptaion									
	Model I					Model II				
	B	SE	β	t	*p*	B	SE	β	t	*p*
(Constant)	2.48	0.65		38.04	<0.001	0.72	0.23		3.10	0.002
Current institution type ^a^	0.07	0.05	0.72	1.33	0.185	−0.01	0.05	−0.01	−0.14	0.885
Shift work ^b^	0.04	0.08	0.30	0.45	0.651	.01	0.07	0.00	0.09	0.926
Satisfaction with current job ^c^	0.48	0.08	0.52	6.11	<0.001	0.30	0.07	0.33	4.26	<0.001
Satisfaction with current job ^d^	0.20	0.06	0.25	3.12	0.002	0.12	0.06	0.15	2.23	0.027
subjective health status ^e^	0.15	0.07	0.14	2.05	0.042	0.06	0.06	0.06	1.00	0.320
subjective health status ^f^	0.06	0.05	0.07	1.06	0.289	0.04	0.05	0.05	0.89	0.372
Turnover intention ^g^	0.25	0.05	0.28	4.74	<0.001	0.15	0.05	0.17	3.26	0.001
Nursing work environment						0.37	0.05	0.38	7.01	<0.001
Nursing practice Readiness						0.32	0.07	0.24	4.68	<0.001
Optimism						0.02	0.04	0.03	0.50	0.616
R^2^	0.420					0.582				
Adj. R^2^	0.399					0.561				
F (*p)*	20.77 (<0.001)					27.55 (<0.001)				

SE: standard error. ^a^ Dummy variable (reference): Hospital (Tertiary general hospital), ^b^ Dummy variable (reference): Shift work (No shift work), ^c^ Dummy variable (reference): Satisfaction with current job (Adequately Satisfaction), ^d^ Dummy variable (reference): Satisfaction with current job (Moderately), ^e^ Dummy variable (reference): subjective health status (Good), ^f^ Dummy variable (reference): subjective health status (Moderate), ^g^ Dummy variable (reference): turnover intention (No intention to leave).

## Data Availability

The data sets used and/or analyzed during the current study are available from the corresponding author upon reasonable request.
